# Using standardized workflows and quantitative data‐driven management to reduce time interval from simulation to treatment initiation

**DOI:** 10.1002/acm2.14284

**Published:** 2024-01-31

**Authors:** Naichang Yu, Danielle LaHurd, Anthony Mastroianni, Anthony Magnelli, Rahul Tendulkar, Samuel T. Chao, John H. Suh, Ping Xia

**Affiliations:** ^1^ Department of Radiation Oncology Taussig Cancer Institute Cleveland Clinic Cleveland Ohio USA

**Keywords:** data‐driven management, standardization, time‐to‐treatment initiation, workflow

## Abstract

**Purpose:**

External beam radiotherapy is a complex process, involving timely coordination among multiple teams. The aim of this study is to report our experience of establishing a standardized workflow and using quantitative data and metrics to manage the time‐to‐treatment initiation (TTI).

**Methods and materials:**

Starting in 2014, we established a standard process in a radiation oncology‐specific electronic medical record system (RO‐EMR) for patients receiving external beam radiation therapy in our department, aiming to measure the time interval from simulation to treatment initiation, defined as TTI, for radiation oncology. TTI data were stratified according to the following treatment techniques: three‐dimensional (3D) conformal therapy, intensity‐modulated radiotherapy (IMRT), and stereotactic body radiotherapy (SBRT). Statistical analysis was performed with the Mann–Whitney test for the respective metrics of aggregate data for the initial period 2012– 2015 (PI) and the later period 2016–2019 (PII).

**Result:**

Over 8 years, the average annual number of treatments for PI and PII were 1760 and 2357 respectively, with 3D, IMRT, and SBRT treatments accounting for 53, 29, 18% and 44, 34, 22%, respectively, of the treatment techniques. The median TTI for 3D, IMRT, and SBRT for PI and PII were 1, 6, 7, and 1, 5, 7 days, respectively, while the 90th percentile TTI for the three techniques in both periods were 5, 9, 11 and 4, 9, 10 days, respectively. From the aggregate data, the TTI was significantly reduced (*p* = 0.0004, *p* < 0.0001, *p* < 0.0001) from PI to PII for the three treatment techniques.

**Conclusion:**

Establishing a standardized workflow and frequently measuring TTI resulted in shortening the TTI during the early years (in PI) and maintaining the established TTI in the subsequent years (in PII).

## 1 INTRODUCTION

One of six aims identified by the Institute of Medicine for the 21st century is timely treatment and reduction of treatment delays, which can be detrimental for both patients and caregivers.^1^ A systematic review by Chen et al. on the relationship between the waiting time for radiotherapy and clinical outcomes^2^ found that the risk of local recurrence increases with the increasing time interval to begin radiotherapy, which in turn may result in decreased survival for breast and head‐and‐neck (H&N) cancers. In the Netherlands, the maximum interval between the diagnosis to treatment for patients with H&N cancer is targeted to be no more than 30 days, but this goal was met only for 34% of patients with H&N cancer between 1990 and 2011.^3^ Using the National Cancer Database (NCDB) from the United States, Murphy et al. studied the trends and predictor of the time‐to‐treatment initiation (TTI) for a patient with H&N squamous cell carcinoma between 1998 and 2011.^4^ The overall median TTI for all H&N squamous cell cancer patients was 26 days but increased from 19 days in 1998 to 30 days in 2011, a 58% increase. From the National Cancer Database from the United States, Khorana et al. studied TTI and its association with the survival of patients with early‐stage breast, prostate, lung, colorectal, renal, and pancreas cancer from 2004 to 2013.^5^ They found that TTI increased 38% over the study period. This rise in TTI can be potentially attributed to the use of sophisticated pretreatment radiological/pathologic investigations, complexity of multimodality therapies, and delays in the transition of care. Starting in 2014, our institution initiated a multidisciplinary program to reduce TTI, achieving a 33% reduction from a baseline median TTI of 29 and 41 days for internally and externally diagnosed patients, respectively.^6^

Radiation therapy is one of three major treatment modalities for many cancer patients. Radiation therapy is a complex undertaking involving physicians, medical physicists, dosimetrists, therapists, schedulers, and nurses coupled with increasingly sophisticated linear accelerators and computer‐based treatment planning. An interruption anywhere in the process may result in a significant delay in treatment initiation, creating unnecessary stress on patients and team members. Therefore, careful coordination among different teams is paramount in reducing TTI. In radiation oncology, it is recognized that a standardized external beam radiation (EBRT) workflow can improve treatment quality, enhance efficiency, and reduce miscommunication or human errors.^7^ Given large variations in available resources and clinical practices, the question raised in a recent debate is how highly standardized and strictly followed a workflow should be.^7^ Both sides of the debate agreed on the standardization of the EBRT process but differed on the standardized time allotted for each step of the EBRT process under many clinical scenarios. The aim of this study is to report our experience of developing a standardized workflow using quantitative metrics to manage simulation to treatment processes while reducing unnecessary delays to initiate radiation treatment. Our hypothesis is that establishing a standardized workflow and frequently measuring TTI can shorten TTI.

## 2 MATERIALS AND METHODS

Since 2014, we have increased awareness of the importance of TTI, started to measure TTI monthly and used TTI as one of the annual key performance indicators (KPIs) for our department. The interventions we implemented over the years were gathering data, measuring TTI, and establishing a standardized workflow that enables accurate measurement of TTI. Due to COVID, our standard operations were interrupted from 2020 to 2022 (e.g., frequent patient treatment delays and cancellations) though we gradually returned to normal practice in 2023.

### 2.1 The standardized workflow

**FIGURE 1:**
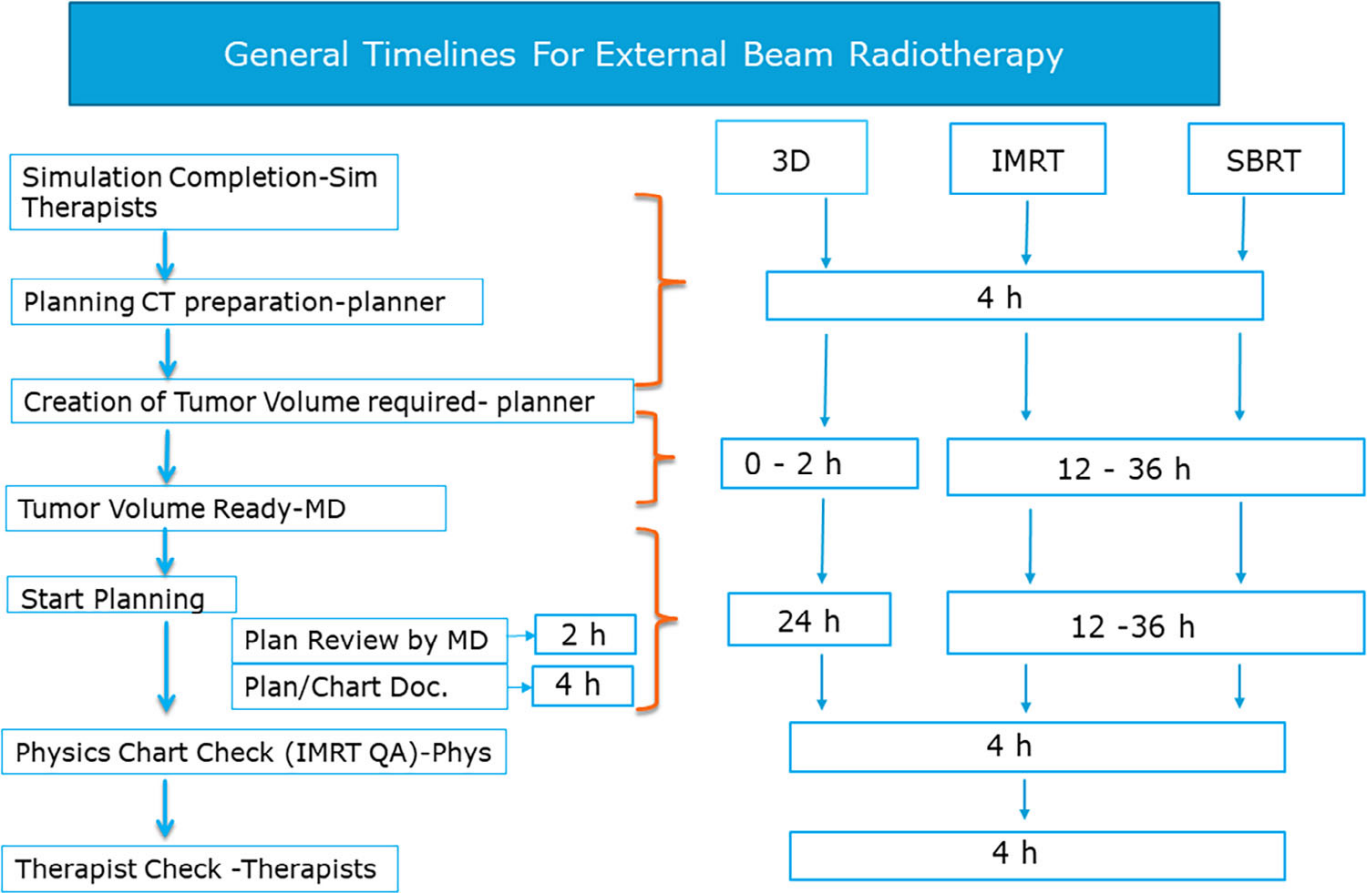
A typical flowchart (on left) and clinical tasks with task owners and general timelines (on the right), defined either in business hours (defined as from 6:00 a.m. to 6:00 p.m.) of major tasks for 3D, IMRT, and SBRT treatments.

For EBRT, in 2014, we established a general workflow chart from the initial consult to the treatment initiation. Briefly, after a clinical consult, if EBRT is recommended, a simulation order from a radiation oncologist is created in our Radiation Oncology Specific Electronic Medical Record (RO‐EMR) system (MOSAIQ, Sunnyvale, CA). Depending on the treatment technique indicated on the simulation order, insurance pre‐authorization may be required for some intensity‐modulated radiotherapy (IMRT) and stereotactic body radiotherapy (SBRT) treatments. If a patient's clinical condition requires urgent or immediate EBRT, a fast‐track (simulation and treatment within 2 days) or an ultra‐fast track (simulation and treatment on the same day) are indicated on the simulation order. For nonurgent patients, a general workflow chart along with a series of clinical tasks and associated task owners is followed as shown in Figure 1.

### 2.2 Timelines and quantitative measures

For IMRT treatment, the timestamps for completing the simulation, image fusion, tumor volume delineation, treatment planning, physician plan review, plan document preparation, physician approval, physics check, and IMRT QA are individually tracked (see Section 2.3 below for details). For each major task, the timestamp of either the completion of a task or the approval of a document is used for tracking task completion. The timelines for completion of these major tasks are standardized at our institution and specified in our standard operating procedures. Figure 1 also shows the typical timelines of major tasks for 3D, IMRT, and SBRT treatments in business hours, which are defined as from 6:00 a.m. to 6:00 p.m., excluding weekends and holidays. In the remaining text, hours refer to the business hours. Upon completion of the simulation, planning CT images and site‐specific contour templates are prepared by dosimetrists or medical physicists in the MIM software (version 6.9, MIM, Cleveland, OH), a program we use for contouring and image fusion. If image fusion is requested in the simulation order form, the medical physics and dosimetry group should complete the task within 4 h from the completion of the simulation. Depending on the complexity of treatment sites and the clinical schedules of radiation oncologists, the targeted contouring time varies from 12 to 36 h, including tumor volumes and critical organs. The planning time includes a plan review with radiation oncologists and plan documentation. The physics chart check time for one chart is typically 15−45 min, depending on the complexity of the plan. Assuming more than one chart is on the task list for a physicist assigned to plan/charts check, the targeted physics plan/chart check time is 4 h. IMRT QA is typically done after business hours, which is not tracked. The goal is to have a “Therapist Check” task created 4 business hours before the first appointment on the treatment machine thereby allowing adequate time for therapists to conduct their review of the patient chart and prepare for the first day of treatment.

### 2.3 QMAP program and statistical analysis

For patients receiving EBRT at the main campus of our institution from 2012 to 2019, TTI data, defined as from simulation to treatment initiation for this specific study, was extracted from our RO‐EMR system using an in‐house developed quantitative metric and automated auditing program (QMAP).^8^ From our RO‐EMR system, QMAP automatically captures monthly treatment volume and the completion timestamp of each clinical task for new patients and patients under treatment. The QMAP system also provides frequent alerts of potential delinquency, including pending tumor volume contour completions, pending imaging reviews, and pending weekly physics quality checks. Within a predefined time window that would offer an opportunity to mitigate the potential for late completion, these alerts are sent to designated triage teams via emails and also posted in the internal website. QMAP can also proactively measure time intervals between any intermediate steps and provide warning reports for pending tasks outside of the targeted time windows. TTI, rather than the time interval from the physician consult to the treatment initiation, is a parameter better controlled by our internal workflow and resources independent of external factors (e.g., insurance authorization). Depending on the treatment complexity, TTI data were stratified by planning techniques of 3D, conventional fractionated IMRT (including volumetric modulated arc therapy––VMAT), and SBRT (planned with either IMRT, conformal arc, or VMAT). Statistical analysis was performed with the Mann–Whitney test for the respective metrics of aggregate data for the initial period 2012 to 2015 (PI) and the later period 2016−2019 (PII). As mentioned above, our initial awareness of TTI started in 2014, and some time was required to implement the standardized process and have each of our team members adhere to the process. Thus, we chose to define the years of 2014−2015 as the transition years while the years of 2016−2019 were considered as a stable period of performance.

## 3 RESULTS

**FIGURE 2:**
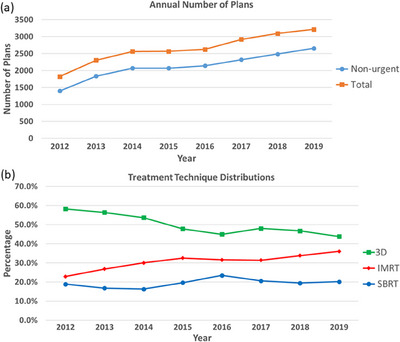
(a) The annual number of new plans from 2012 to 2019, separating the nonurgent plans from the total annual plans. (b) The plan distributions from 2012 to 2019 among three different treatment techniques: three‐dimensional conformal plans (3D), intensity modulated radiotherapy (IMRT) plans, and stereotactic body radiotherapy (SBRT) plans.

Figure 2a shows the total number of new plans from 2012 to 2019 for the main campus of our institution (data pulled from the QMAP system). The ratio of urgent plans and nonurgent plans was 20%/80% and has remained stable over the years. Figure 2b is the plan distributions among three different treatment techniques: 3D, IMRT, and SBRT plans (data pulled from the QMAP system). Patients treated with Gamma Knife radiosurgery were excluded from this study. As shown from Figure 2a,b, the number of new patients increased 7.7% per year, of which the percentage of patients treated with 3D decreased and patients treated with IMRT and SBRT increased, indicating a gradual increase in planning complexity.

**FIGURE 3:**
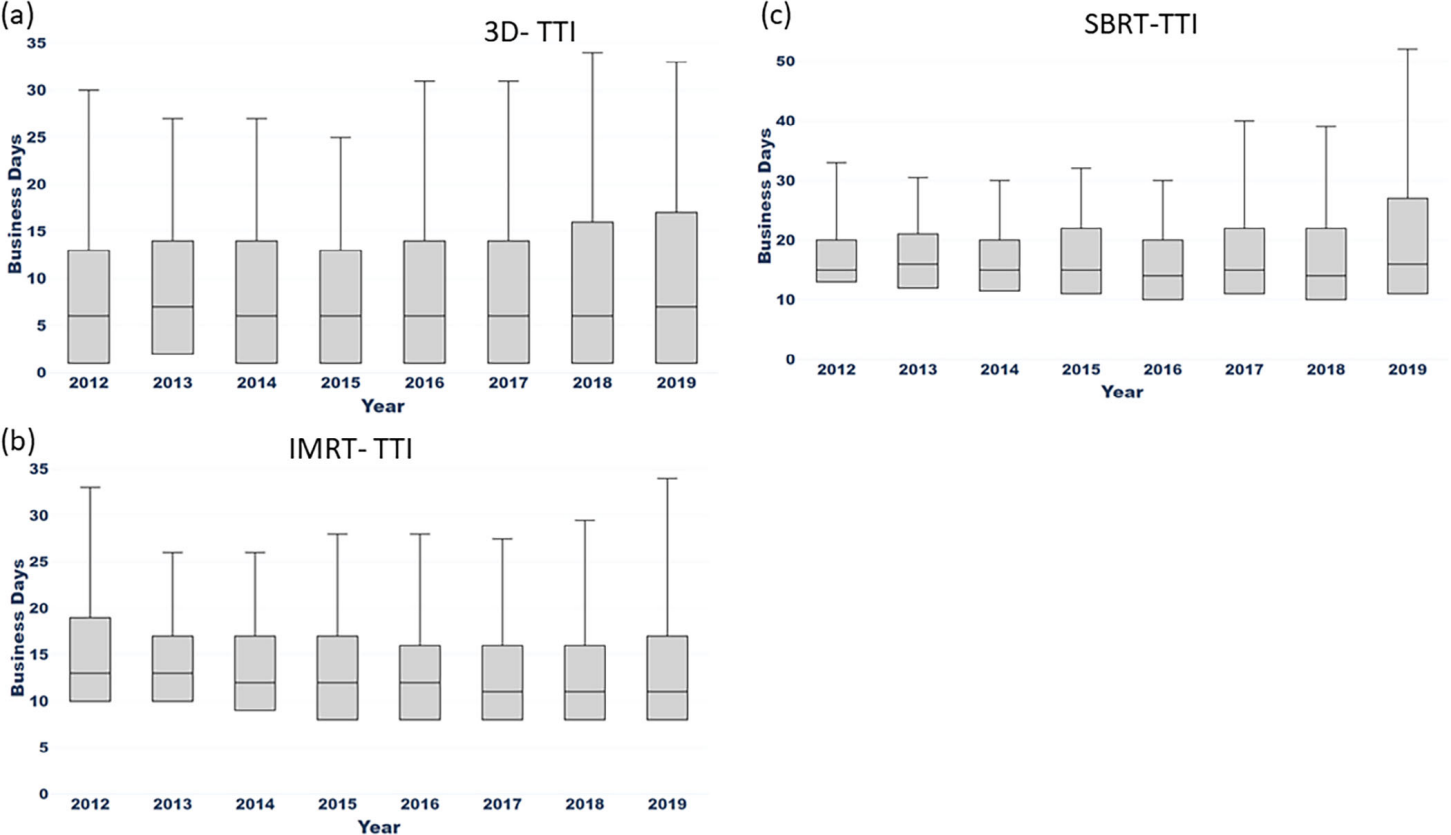
Panels (a–c) are the box plots of TTI for three typical treatment modalities of 3D, IMRT, and SBRT. TTI, simulation to treatment initiation.

As shown in Figure 3a, the median TTI time interval for 3D plans is very short, about 1 day, including 20% of the urgent plans. Of note, most 3D plans were simple and did not require detailed contours of tumors and organs‐at‐risks. Statistical analysis of the aggregated data from 2012 to 2015 and from 2016 to 2019 showed TTI of the 3D technique was reduced (*p* = 0.0004) from PI to PII. The median TTI and 90th percentile of the 3D technique were 1, 5 days in PI and 1, 4 days in PII.

For the IMRT technique, statistical analysis of the aggregated data showed that the TTI was significantly reduced (*p* < 0.0001) from PI to PII. The median TTI of IMRT was reduced from 6 days in PI to 5 days in PII while the 90th percentile remained stable at 9 days over the years. For the SBRT technique, statistical analysis of the aggregated data showed that the TTI was significantly reduced from PI to PII (*p* < 0.0001). The median TTI and 90th percentile of SBRT were 7, 11 days in PI compared to 7, 10 days in PII.

**FIGURE 4:**
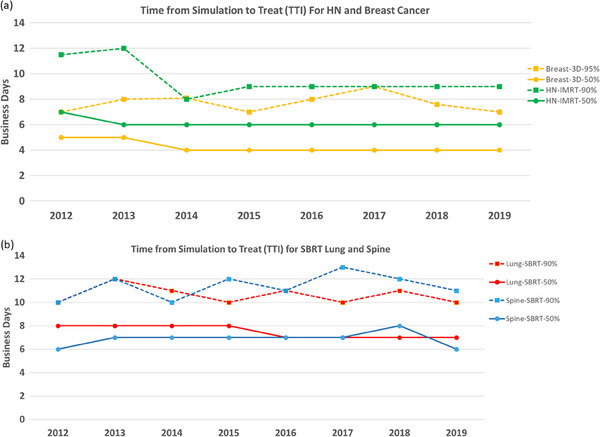
(a) The median and 90th percentile of time from the simulation to treatment initiation (TTI) for HN and breast cancers; (b) the median and 90th percentile of time from the simulation to treatment initiation (TTI) for lung SBRT and spine SBRT procedures.

Using breast cancer as an example for nonurgent 3D plans, Figure 4a shows the median TTI time interval decreased from 5 days in PI to 4 days in PII. Since the median time interval only applies to the 50th percentile of patients, we chose an additional data point of the 90th percentile TTI. As shown in Figure 4a, after simulation, 90% of our breast patients started their radiation treatment in 7 days in 2018−2019, decreased from 8 days in 2013−2014. Statistical analysis of the aggregated data confirmed that the TTI of breast plans was reduced (*p* < 0.0001) from PI to PII.

Using H&N cancer as an example for IMRT plans, Figure 4a also shows the median TTI time interval of 6 days for most years. IMRT plans for H&N cancer are known for planning complexity, involving many organs‐at‐risk, and complex tumor volumes with 2−3 different prescription dose levels. Prompted by our priority to shorten time‐to‐treatment, for 90% of H&N patients, Figure 4a shows a noticeable decrease of the 90th TTI from a maximum 12 days in PI to a sustainable 9 days in PII. It is noted that there was a decrease in the 90th percentile TTI from 2013 to 2014 prior to formally implementing TTI measures. This reduction can be attributed to adding one more physician to the HN group. Statistical analysis of the aggregated data from 2012 to 2015 and from 2016 to 2019 shows that the TTI of HN plans from the two periods was not statistically different (*p* = 0.86).

Figure 4b shows the median TTI and 90th percentile for TTI for two SBRT sites: spine and lung. For lung SBRT, the median TTI was reduced from 8 days in PI to 7 days in PII while the 90th percentile TTI was reduced from a maximum of 12 days in 2013 to a maximum of 11 days after 2015. Statistical analysis of the aggregated data shows that the TTI of SBRT lung plans from the two periods was statistically reduced (*p* < 0.0005). For spine SBRT, the median TTI was stable over the years, 7 days and the 90th percentile TTI was increased from a maximum of 12 days in PI to a maximum of 13 days in PII. Statistical analysis of the aggregated data showed that the TTI of SBRT spine plans was not significantly different from the two periods (*p* = 0.85).

## 4 DISCUSSION

With careful workflow designs, care team coordination, and frequent quantitative data measurements, we demonstrated the ability to establish a standardized process and stable time intervals for TTI (defined as a simulation to treatment initiation) for different treatment modalities that include various levels of technical difficulties. As stated in a report from the Institute of Medicine on Delivery of High‐Quality Cancer Care, cancer care is extraordinarily complex, with many specialities involved in the routine diagnostic and therapeutic planning of care (e.g., radiology, pathology, surgery, medical and radiation oncology, nursing).^9^ Delivery of quality care requires coordination among these disciplines. The process of radiation therapy is also complex, also involving coordination among physicians, nurses, dosimetrists, medical physicists, and radiation therapists while using modern linear accelerators and computer‐based treatment planning systems. Under the leadership of our cancer center and radiation oncology department, our team members have a mission to provide high‐quality cancer care and understand that providing timely cancer treatment is an important metric. The concept of KPIs was proposed in 2013^10^ and this concept is now being introduced in clinical practice.^11^ The question is how to identify specific and meaningful KPIs in radiation oncology. We suggest that TTI, time from consult to simulation, and time from simulation to treatment initiation are meaningful KPIs.

Given public scrutiny regarding safety in radiation therapy, highlighted by a few published catastrophic safety incidents, we must carefully balance safety issues when determining a reasonable TTI to allow each team to complete their critical clinical tasks. In this study, depending on the complexity of treatment planning and delivery, we used data over a span of 8 years to define a reasonable TTI time interval for each treatment modality. In response to the call of improving radiation safety,^12^ many major academic centers reported their strategies to prevent safety incidents. Some of these reports identified that rushed processes were associated with a higher risk of radiation incidents while others found that there was no relationship between time spent in radiation planning and safety events. Walker et al. from The University of Texas MD Anderson Cancer Center identified that complicated plans, fewer fractions, first day of treatment, and rushed processes were associated with a higher risk of radiotherapy incidents.^13^ They defined the number of prescription items in a plan as a surrogate for plan complexity. Among treatment planning errors (any errors in the treatment plans that can lead to mistreatments [e.g., dosimetric errors]), they found that less time from plan approval to treatment start was associated with a higher rate of planning errors. We recognize the importance of post‐plan checks from medical physicists and radiation therapists and have carefully measured our late chart metric monthly.^8^ After reviewing their 10‐year trends in safe radiation therapy delivery, Dominello et al. found that a rushed environment was associated with an increase in replanning due to a treatment unit being down.^14^ Their strategy to avoid a rush environment was to only replan selected patients, as determined by attending physicians.^14^ Our strategy to avoid rush environment issues is to have at least two matched treatment machines. We understand that this strategy may not be applicable to all centers, notably for centers with a single treatment machine. Gensheimer et al. examined the influence of planning time and treatment complexity on radiation therapy errors in 2257 treatment courses.^15^ When controlling for treatment technique and other clinical factors, they found that there was no relationship between time spent in radiation planning and near‐miss events. Specifically, they found that TTI for IMRT and SBRT was not predictive of near‐miss events on univariate or multivariate regression. As for differences in incident rates among various cancer treatments, Elnahal et al. further identified patients with H&N and breast primaries had higher incident rate than patients treated with brain lesions.^16^ They also identified that patients with large tumors and treated with IMRT/IGRT were more likely to have errors that deviate from the intended treatment. These identified patient factors, allotments of adequate planning time, and planning processes may be associated with potential medical incidents (defined as either reportable medical events or deviations from treatment intents) but conclusions from these studies may not be easily generalized since the resources and processes from different institutions can be drastically different. While the purpose of our study was not to identify risk factors of medical incidents, minimizing TTI may potentially contribute to a rushed environment, increasing the medical incident rate.

One limitation of this study is that we used a single institution data, which may not be generalized to other departments due to the different resources and clinical environments. Another limitation of the study is that the TTI data did not directly correlate to any specific causal factor. We believe that using quantitative data of TTI allows us to identify potential bottlenecks in our workflow while providing additional resources if necessary and frequent measurements of TTI promote awareness and motivate behavior changes.

## 5 CONCLUSIONS

High‐quality cancer care should be designed with careful considerations of timeliness of care, coordination among multiple disciplines, and allocation of adequate time for each team to carry out the clinical tasks. As noted, a rushed process often reflects a poorly coordinated process. Carefully measuring the required time for each step in the entire radiation therapy process allows us to define the appropriate timelines from simulation to treatment initiation based on the complexity of the treatment process. This measure can also allow us to allocate needed resources such as adding additional physicians or implementing AI‐based contouring tools for normal critical organs. In conclusion, the development and implementation of data‐driven management tools systematically decrease or stably maintain the established TTI, which improves patient access to cancer care.

## AUTHOR CONTRIBUTIONS

All authors listed contributed to the content of this paper including study design, data collection, data analysis, and writing/editing the manuscript.

## CONFLICT OF INTEREST STATEMENT

The authors declare no conflicts of interest.
